# Prevalence of adverse childhood experiences and their relationship to mental and physical illnesses in the Eastern Region of Saudi Arabia

**DOI:** 10.1002/brb3.2668

**Published:** 2022-06-27

**Authors:** Amena Hamood AlHemyari, Nada Adeeb Al‐Zamil, Ahad Yasir Shaikh, Dalal Abdulaziz Al‐Eidi, Hussam Waleed Al‐Dahlan, Sumiyah Sulaiman Al‐Shamekh

**Affiliations:** ^1^ Department of psychiatry, Imam Abdulrahman Bin Faisal University Dammam Saudi Arabia; ^2^ Ministry of Health Dammam Saudi Arabia; ^3^ Department of psychiatry, King Saud University Medical City (KSUMC) Riyadh Saudi Arabia

**Keywords:** ACEs, adverse childhood events, chronic disease, mental health, trauma

## Abstract

**Introduction:**

Adverse childhood experiences (ACEs) are widely prevalent and interrelated. They affect multiple domains of health while having a dose–response effect. These effects are biologically plausible, where ACEs are found to be highly associated with physical and mental comorbidities.

**Objectives:**

The study aimed to measure the magnitude of ACE and its relationship to mental and physical illnesses in the Eastern Region of Saudi Arabia by assessing its prevalence.

**Materials and Methods:**

This was a retrospective cohort study that took place in the Eastern Region of Saudi Arabia in 2020.

**Setting:**

It was applied to a population‐based, random adult sample from both genders, different educational levels and socioeconomic statuses.

**Participants:**

Those who were 19 years old and above and living in the Eastern region were included. Everyone under the age of 19, those not currently living in the Eastern Region, and those who did not complete the questionnaire were excluded.

**Results:**

The total sample size was 611 respondents, but after applying the exclusion criteria, 507 respondents were included. Most participants were females (65.1%). The mean age of the participants was 29.7 years, with a standard deviation of 11.2 years. Regarding educational level, 69.6% were college/university graduates. Most study respondents (81.8%) were exposed to four or more types of ACEs, with emotional neglect being the most common type (82.2%). Having four or more ACEs increases the risk of having physical illnesses compared to those with only one. Furthermore, female respondents who had four or more ACEs had the highest likelihood of having depressed mood (Adjusted odds ratio [AOR] = 1.04; 95% confidence interval [CI] = 1.0–1.07), stress (AOR = 2.8; 95% CI = 1.11–7.3), and insomnia (AOR = 1.04, 95% CI = 1.01–1.07).

**Conclusion:**

Our study showed that in the Eastern Region, ACEs are highly prevalent and are associated with an increased risk of mental and physical illness.

## Graphical Abstract

Graphical abstract

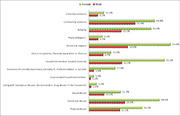

## 1 INTRODUCTION

There is growing interest that has developed over the last decade concerning the effect of adverse childhood events on mental and physical well‐being (Van duin et al., 2019). Adverse childhood experiences (ACEs) have been described as potentially traumatic events that are capable of having a lifelong effect on one's health and general well‐being (Moffitt, 2013). ACEs can be categorized into three main types: abuse, neglect, and household dysfunction (Boullier & Blair, 2018; Chang et al., 2019). ACEs adversely affect physiological functions by inducing a state of chronic toxic stress, which is correlated with changes in our molecular and genetic makeup during childhood (Nurius et al., 2015; Su et al., 2015). Toxic stress was found to be associated with dysfunction of the allostatic systems, which include the nervous, endocrine, and immune systems (Boullier & Blair, 2018; Oral et al., 2016). ACEs have been found to increase the risk of chronic diseases (Anda et al., 2010, 2008), mental illnesses (Anda et al., 2002; Bebbington et al., 2011), and premature death (Anda et al., 2009; Felitti et al., 1998; Holter et al., 2020; Merrick et al., 2017). Moreover, violence and substance abuse are strongly related to ACEs (Alhowaymel et al., 2021; Anda et al., 1999; Mersky et al., 2013; Nurius et al., 2015). Such factors can lead to ACEs in the next generation in the form of parental domestic violence, mental illnesses, and substance abuse, locking families into a cycle of poor well‐being (Hughes et al., 2017). Overall, the prevalence of participants reporting at least one ACE in Saudi Arabia was 87.7%, and 49.2% reported four or more ACEs (alhowaymel, 2020). Moreover, we postulate that public awareness regarding the deleterious effects of ACEs on long‐term health needs to be raised since corporal punishment and other forms of abuse are still socially accepted by some parents in the Middle East. Since there is a scarcity in the studies of the prevalence of ACEs in the Eastern Region of Saudi Arabia, we believe that studying such important risk factors can help identify the magnitude of the problem and eventually design programs to target children at risk and provide them with the necessary psychosocial resources early on to reduce long‐term complications.

## 2 PURPOSE

### 2.1 General objective

This study aimed to assess the prevalence of ACEs in the Eastern Region of Saudi Arabia.

### 2.2 Specific objectives

The specific objective of this study was to investigate the relationship between ACEs and physical and mental illnesses.

### 2.3 Methods

*Study design*: This is a retrospective cohort, questionnaire‐based study evaluating the prevalence of ACEs and their relationship with physical and mental illnesses. This study was approved by Imam Abdulrahman bin Faisal University's institutional research board (IRB) ethical committee. It is considered a retrospective cohort study because we are measuring the prevalence of ACEs’ impact on mental and physical health throughout adulthood.

### 2.4 IRB approval number IRB–UGS‐2020‐01‐195

*Setting and participants*: The sample included 611 participants who were randomly selected, including both genders with various educational levels and different socioeconomic statuses by distributing the questionnaire on different platforms.

The inclusion criteria are the age of 19 years old and older (because in our country people tend to use both the Hijri and Gregorian colanders and that has some discrepancies in ages) and residing in the Eastern province of Saudi Arabia. Participants who did not meet these criteria and those who refused to fill the questionnaire or incomplete the questionnaire were excluded. This study was conducted through an electronic survey distributed through social media platforms and sent by email to all Imam Abdulrahman bin Faisal University (IAU) employees and students. Data were collected over 4 months, from June 2020 to September 2020. All participants agreed to participate voluntarily and answer the questionnaire truthfully and anonymously. After applying the inclusion criteria, 104 responses were excluded, 62 respondents were not living in the Eastern Province, 22 incomplete questionnaires, and 20 respondents were less than 19 years old.

*Materials*: ACEs were evaluated using the Adverse Childhood Experiences International Questionnaire (ACE‐IQ), which is a validated screening instrument that was developed by the World Health Organization to measure ACEs in all countries and the association between them and risk behaviors in later life.

In our study, we used the ACE‐IQ to assess exposure to 13 types of ACEs, including emotional abuse, physical abuse, sexual abuse, violence against household members, living with household members who were substance abusers, living with household members who were mentally ill or suicidal or imprisoned, having one or no parents, parental separation or divorce, emotional neglect, physical neglect, bullying, and community and collective violence. The English version of the ACE score calculator was translated into Arabic by the researchers, and cross‐cultural adaptation was carried out through an iterative forward‐backward translation compared to an independent third person.

The Cronbach's alpha for the ACE‐IQ questionnaire as a total for the current study was 0.875, which indicates sufficient internal consistency.

To screen for physical and mental symptoms in the sample, we used the health appraisal questionnaire developed by the Centers for Disease Control and Prevention , which has two versions: male and female. Certain questions from the male and female health questionnaires were removed due to a low response rate secondary to cultural reasons. The Cronbach's alpha for the health appraisal (male and female versions) was 0.833 and 0.872, respectively, which is high in reliability (>0.7), indicating that both domains were valid and reliable.

*Procedure*: This study was conducted through an electronic survey distributed through social media platforms and sent by email to all IAU employees and students. Data were collected over 4 months, from June 2020 to September 2020. All participants agreed to the informed consent to participate voluntarily and answer the questionnaire truthfully and anonymously. (At the beginning of the questionnaire, there was a statement of consent explaining that if the participants were willing to answer these questions for research purposes, their information and identity would be confidential. if the participants consented, they can press continue to move on to the questions)

*Data analysis*: The ACE score is calculated by summing all 13 ACE variables and serves as a measure of overall ACE exposure ranging from 0 to 13. Responses were further analyzed according to the number of adverse experiences (1, 2, 3, 4, or more) before 18 years of age. Statistical Package for Social Sciences (SPSS, ver.25.0) software was used for data entry and statistical evaluation. The following statistical methods were used for data analysis:

- descriptive statistical methods,
- correlation,
- chi‐square analysis,
- logistic regression analysis was employed to adjust for potential confounding factors,
- the prevalence of ACEs was determined,
- estimates of odds ratios were computed to measure the association between ACEs and physical and mental health.

*p*‐Values below .05 were considered statistically significant in all statistical analyses.

## 3 RESULTS

The total sample size after applying the exclusion criteria was 507, with the majority of participants being females, with a percentage of 65.1%. The mean age of the participants was 29.7 years, with a standard deviation of 11.2 years. Note that 69.6% of our participants were university graduates, while more than half of our sample size were single, representing 52.3%. Table 1 summarizes the sociodemographic data.

**TABLE 1:** Demographic profiles of respondents (*n* = 507)

	No.	Percentage (%)
Gender		
Male	177	34.9
Female	330	65.1
Age group (years) ^a^		
<20	58	11.5
20–29	274	54.5
30–39	72	14.3
40–49	53	10.5
50–59	37	7.4
60+	9	1.8
Marital status		
Married (go to Q. M2)	188	37.1
Living as couple	27	5.3
Divorced or separated	15	3.0
Single	265	52.3
Widowed (go to Q. M2)	1	0.2
Other	4	0.8
Refused	7	1.4
Education level		
Primary school completed	1	0.2
Secondary/high school completed	98	19.3
College/university completed	353	69.6
Postgraduate degree	54	10.7
Refused	1	0.2
Work status		
Government employee	86	17.0
Nongovernment employee	103	20.3
Self‐employed	3	0.6
Nonpaid	5	1.0
Student	228	45.0
Homemaker	26	5.1
Retired	21	4.1
Unemployed (able to work)	31	6.1
Unemployed (unable to work)	3	0.6
Refused	1	0.2

^a^ 4 (0.8%) missing data.

### 3.1 Prevalence of ACEs in the Eastern Region

**FIGURE 1:**
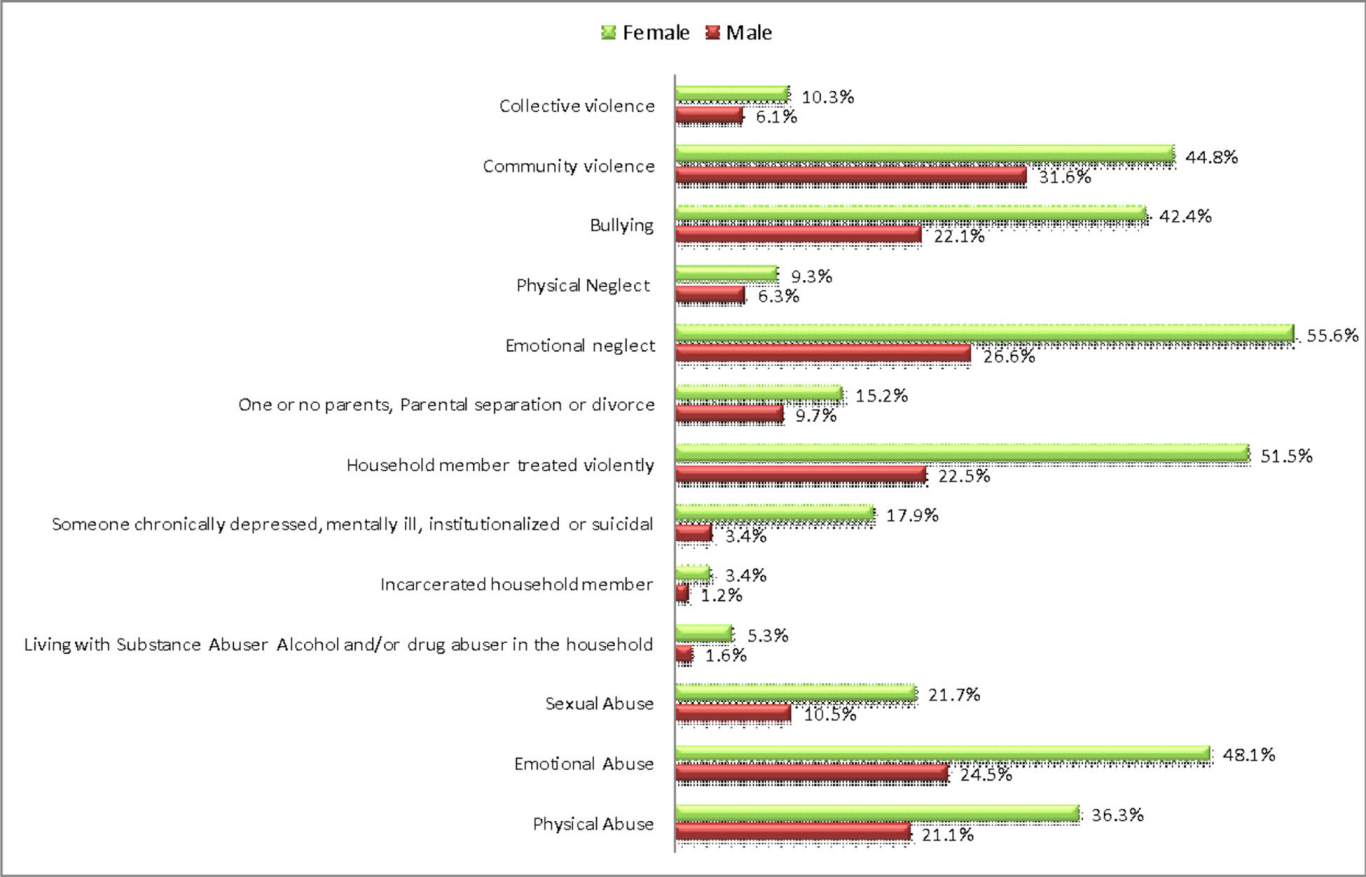
Prevalence of adverse childhood experiences (ACEs) by gender

After analyzing the data, the results revealed that the most reported ACEs (Figure 1) were emotional neglect (82.2%), followed by community violence (76.3%), witnessing household members treated violently (74%), emotional abuse (72.6%), bullying (64.5%), physical abuse (57.4%), sexual abuse (32.1%), living with one or no parents (24.9%), mentally ill household member (21.3%), collective violence (16.4%), and physical neglect (15.6%).

Moreover, 10% of respondents reported living with substance or alcohol abusers and incarcerated household members, with percentages of 6.9% and 4.5%, respectively. Figure 1 summarizes the prevalence of all 13 ACEs.

### 3.2 ACE‐IQ score

The average score for the binary method of scoring was 5.49, with a range of 1–13. As shown in Table 2, 3.4% had at least one ACE. A total of 6.5% and 8.3% of participants reported experiencing two and three ACEs, respectively. However, most study respondents (81.8%) were exposed to four or more types of ACEs (Chartier et al., 2010).

**TABLE 2:** Adverse childhood experiences (ACE) scores

ACE‐IQ score	No.	Percentage (%)
0	–	–
1	17	3.4
2	33	6.5
3	42	8.3
≥4	415	81.8
Total	507	100.0
Average score	5.49	
SD	2.23	
Range	13	

### 3.3 ACE prevalence in the Eastern Region by gender

Female respondents were exposed to ACEs more than males. Hence, the prevalence of ACEs was significantly higher among females (*p* < .001), mainly having someone chronically depressed, mentally ill, institutionalized, or suicidal, witnessing household members treated violently, and experiencing emotional neglect and community violence (Figure 1).

### 3.4 Abuse

Questions of abuse prevalence were directed toward physical, emotional, and sexual abuse among male and female responders, with the prevalence of physical abuse being 21.1% and 36.6%, while emotional abuse was reported by 24.5% and 48.1% and sexual abuse was reported by 10.5% and 21.7%. There was no statistically significant difference between genders in all types of abuse.

### 3.5 Household dysfunction

The results showed that 6.9% of participants reported living with grown‐up with substance or alcohol use disorders, and 4.5% had a member of their household incarcerated. The prevalence of having a family member who is chronically depressed or mentally ill was 21.3%, with a significant difference found between genders: 3.4% among male participants and 17.9% among female participants. While 74.0% of our participants had a family member who was treated violently, there was a significant difference between genders, in which the percentage of females was 51.5%. Moreover, there were significant differences regarding the following questions: Did you see or hear a parent or household member in your home being yelled at, screamed at, sworn at, insulted, or humiliated? and did you see or hear a parent or household member in your home being slapped, kicked, punched or beaten up?; it was also statistically higher among female respondents. A total of 24.9% of our participants reported divorce or separation of parents, with no difference between genders.

### 3.6 Neglect

A total of 82.2% of participants reported emotional neglect (26.6% and 55.6% among males and females, respectively), while 15.6% reported physical neglect, with no statistically significant difference between genders.

### 3.7 Health appraisal questionnaire results

Regarding the prevalence of physical health conditions among the participants, the results showed that the most reported health condition was high blood pressure (19.5%), with a higher prevalence in females than in males. Obesity was the second most common condition (19.3%) with female predominance. A total of 14.8% of the participants reported kidney and bladder diseases with no significant sex differences. The least common physical condition was diabetes mellitus (5.5%), which can be explained by the young age of the majority of participants, which was highly prevalent among male participants.

Regarding mental health conditions, there was a female predominance in most of the reported symptoms. The most prevalent symptom was tiredness (60.7%), followed by depressed mood (60.6%). Insomnia and crying spells were reported by 58.4% and 41.6%, respectively, and were significantly prevalent among females (*p* < .05, *p* < .001, respectively). Although 19.7% reported feelings of stress and anxiety with a female predominance as well, there was no statistically significant difference between genders.

For the health risk behaviors, our questions targeted the following: smoking, recreational drug use, harmful alcohol use, and not using seat belts. The prevalence of smoking was 20.9%, and it was significantly higher among males. Twenty percent of the respondents reported not using seat belts, which was significantly higher among female participants (*p* < .001). Furthermore, harmful alcohol use was reported by 1.2%, while the prevalence of recreational drug use was 0.8%, with no significant difference between genders.

### 3.8 The relationship between ACEs and adulthood physical and mental illnesses

The relationship between different ACE scores and physical and mental health was examined using logistic regression analysis. It was noticed that having four or more ACEs increases the risk of having physical illnesses in comparison to those with one ACE. Furthermore, female respondents who were exposed to four or more ACEs had the highest likelihood of having depressed mood (Adjusted odds ratio [AOR] = 1.04; 95% confidence interval [CI] = 1.0–1.07), stress (AOR = 2.8; 95% CI = 1.11–7.3), insomnia (AOR = 1.04, 95% CI = 1.01–1.07), and crying spells (AOR = 1.05, 95% CI = 1.01–1.09).

## 4 DISCUSSION

This study provides the Kingdom of Saudi Arabia (KSA) population‐based prevalence estimates of the presence of ACEs in a nonclinical sample. An additional purpose of the current study was to determine whether ACEs were associated with a greater prevalence of physical and mental health consequences. We hypothesized that an increase in ACEs would be associated with a higher rate of physical and mental comorbidities. The results indicate that the presence of one or more adverse experiences in childhood was associated with a higher likelihood of having physical and mental illnesses compared to those who experienced none.

Our prevalence estimates are 72.6% for at least one ACE. These estimates are higher than those demonstrated in previous national studies (52%) and international studies (5%–34.4%). When comparing the prevalence rate to the current literature, this study demonstrated a markedly elevated estimate of ACEs (Table 3a,b). In addition, the prevalence of physical abuse in this study was found to be 57.4%, which corresponds closely to the national study (42%); however, it was much higher than international rates of 1.3%–17.9%. The prevalence of sexual abuse was found to be 32%, which is also higher than the prevalence in a previous national study (21%) (Al‐eissa et al., 2016; Almuneef et al., 2018). Previous international studies showed that females were more exposed to emotional, physical, and sexual abuse than males (Subramaniam et al., 2020).

**TABLE 3:** Compaires the results in the currunt study versus previous studies conducted in Saudi Arabia (a), and Internaionally (b)

(a) Statistics in the current study versus previous studies conducted in Saudi Arabia		
	Current study (Eastern Region of Saudi Arabia) (%)	Saudi Arabia^4^ (all regions) (%)
Emotional abuse	72.6	52
Physical abuse	57.4	42
Sexual abuse	32.1	21
Household substance abuse	6.9	9
Household mental illness	21.3	10

(b) International statistics							
	United States^25^	United States^26^	Philippine^27^	Iraq^28^	Hungary^29^	Brazil^30^	Tunisia^31^
Emotional abuse	–	34.4%	22.8%	6.27%	5%	–	–
Physical abuse	–	17.9%	1.3%	17.21%	5%	7%	44%
Sexual abuse	–	11.6%	5.2%	7.52%	1%	1.4%	21%
Household substance abuse	9%	27.6%	36.2%	3.24%	11%	–	9%
Household mental illness	8%	16.5%	6.2%	–	5%	–	10%

In terms of household dysfunction dimensions, we estimated higher rates in terms of living with mentally ill household members (21.3% versus 5%—16.5%). The only dimension in which our sample scored lower was living with a substance abuser (6.9% versus 9%—36.2%). (AlShawi et al., 2019; ; El Mhamdi et al., 2017; Hughes et al., 2017; Merrick et al., 2018; Ujhelyi et al., 2019; Sacks & Murphey, 2018; Soares et al., 2016). The variability in the prevalence of ACEs in our study compared to international rates may be the result of different methodological approaches, such as sample selection. An additional plausible explanation would be the sociocultural factors in which some forms of verbal and physical abuse are still considered socially acceptable methods of discipline by some parents in the Middle East. Furthermore, alcohol and substance abuse are legally and religiously prohibited nationally, which explains the lower rate compared to international rates. While there may be variations in alcohol consumption rates in the KSA compared to international rates, alcohol use may be underreported in the KSA due to social and religious stigma.

This study provides supporting evidence regarding the relationship between ACEs and resulting physical illnesses. There is a significant increase in this relation among females who experienced four or more types of ACEs in comparison with those with at least one ACE type, meaning that the quantity of ACEs correlates with the quantity of physical illness, as previously demonstrated in the literature (Kerker et al., 2015; Riedl et al., 2020). A total of 19.5% reported having high blood pressure, 19.3% obesity, 14.8% urinary tract diseases, and 5.5% diabetes. These results resembled the result reached by Almuneef et al. (2016) in which 21% of their sample reported having hypertension, 15% had obesity, and 9% had diabetes or depression (Almuneef et al., 2016).

Our data show that respondents who were exposed to four or more ACEs had the highest likelihood of mental illness symptoms such as tiredness (60.7%), followed by depression (60.6%), and the least reported was anxiety (19.7%). Almuneef et al. (2016) demonstrated that anxiety was the most prevalent (17%) (Alhowaymel et al., 2021). Female respondents who were exposed to four or more types of ACEs had the highest likelihood of depression. This finding was consistent with previous national study results (AlMuneef et al., 2016). Similar findings were found in England, where there was a strong association between all markers of low mental well‐being and ACE score (Hughes et al., 2017). In Iraq, a statistically significant association between most forms of ACEs and depression was found.

These results should be taken into account considering how experiencing at least one ACE can impact health by resorting to risky health behaviors such as smoking (20.9%), not using seatbelts (20%), alcohol consumption (2.1%), and illicit drug use (0.8%). The results of this study are broadly in line with those of Almuneef et al. (2016), where smoking prevalence was 37.5%. Bellis et al. (2014) found that 11.9% of binge drinking was attributed to ACEs in their study, 58.7% to heroin/crack cocaine use, and 22.7% to smoking (Kim, 2017). Interestingly, Dube et al. (2003) found that each ACE increased the likelihood of early initiation of alcohol by two to fourfold.

The correlation between ACEs and the physical health of respondents appears to be significantly increased among females who experienced ≥4 types of ACEs. Similar studies in Saudi Arabia comparing nonabused participants to abused individuals during childhood showed a twofold increased risk of developing physical health issues such as hypertension, diabetes mellitus, coronary heart disease, and obesity (AlHowaymel, 2020; AlMuneef et al., 2016; Herzog & Schmal, 2018; Lui et al., 2018). Another large study conducted in the United States found that ACEs were associated with one or more of the following health outcomes: diabetes, heart disease, and functional limitations (Monnat & Chandler, 2015).

Since the questionnaire of this study was sent to all IAU employees and students, the link between ACEs and education/employment is rather inaccurate. However, many studies have found a strong association between childhood maltreatment and poor academic performance and unemployment (Altamimi et al., 2017; Ferfusson et al., 2013; Liu et al., 2013; Metzler et al., 2017; Subramaniam et al., 2020).

All studies found in the literature support that the more ACEs the person experiences, the higher the likelihood of developing physical and mental health illnesses in the future (Brown et al., 2010; Chartier et al., 2010; Currie & Widom, 2010; Hughes et al., 2016; Kalmakis & Chandler, 2015; Widom et al., 2008). This can be in part attributed to the effect of early experiences of adversity on the major allostatic systems secondary to the chronic state of stress (Oral et al., 2016).

### 4.1 Implications

The study adds to our understanding of the relationship between ACEs, poor physical and mental health, and the execution of risky health behaviors. These results should be considered given the impact they have on general health. Since the majority of our responders reported being exposed to four or more ACEs, these results implicate a serious concern about the exposure of ACEs in the Eastern Province of the KSA. ACE exposure was especially higher among females, similar to a previous study (Dobson et al., 2020). This might be because they constitute 65.2% of the participants. Nonetheless, it may also indicate that females in the KSA are more prone to abuse due to social roles and dynamics that are gender bound. We believe that increasing awareness of ACEs and their long‐term sequelae is a critical target for all health ministries and agencies.

### 4.2 Conclusion and recommendations

In the Eastern Region of Saudi Arabia, ACEs are highly prevalent and are associated with an increased risk of long‐term mental and physical illnesses. The results showed a high prevalence of most ACEs and how these adversities can negatively impact both physical and mental well‐being. We recommend establishing national programs to raise awareness of ACEs and their disastrous sequelae, with a special focus on educating and training teachers, policemen, healthcare workers, and social workers to help in the early detection of high‐risk groups and detecting signs of abuse. We also recommend working on primary prevention programs such as providing parenting classes and training. We believe that further research in this field would be very beneficial for future practice and will help improve the overall health of society.

### 4.3 Limitations

The data collected in this study were based on self‐reported events that occurred before the age of 19, which may subject this study to recall bias. Since the study was conducted only in the Eastern Region, other provinces may differ in important ways, particularly in rural areas. We noticed that there was a high rate of refusal to participate in this study, given the conservative culture of the society in Saudi Arabia.

## CONFLICTS OF INTEREST

The authors declare no conflict of interest.

## AUTHOR CONTRIBUTIONS

All authors made substantial contributions to conception, design, acquisition of data, analysis and interpretation of data. They were involved in drafting the manuscript or revising it critically for important intellectual content and gave final approval of the version to be published. Each author has participated sufficiently in the work to take public responsibility for the content and agreed to be accountable for all aspects of the work in ensuring that questions related to the accuracy or integrity of any part of the work are appropriately investigated and resolved.

### PEER REVIEW

The peer review history for this article is available at https://publons.com/publon/10.1002/brb3.2668.

## Data Availability

The data that support the findings of this study are available from the corresponding author upon reasonable request.
